# Supplementary material for: Complex Feline Disease Mapping Using a Dense Genotyping Array
Source: Front Vet Sci. 2022 Jun 16;9:862414. doi: 10.3389/fvets.2022.862414 (PMC9244801; doi:10.3389/fvets.2022.862414)
Supplement: Supplementary file 6 [file Data_Sheet_1.DOCX]

Supplementary Material

**S1 Figure**. **LD plots for the GWAS positive controls, using the LMM GWAS results.** (A) *Orange* locus GWAS, showing the significant association on chromosome X. (B) Factor XII deficiency GWAS, showing the A1 significant association in the vicinity of the gene *F12.* Colors indicate the amount of LD (r^2^) with the most significant SNP, ranging from black (r^2^<0.2) to red (r^2^>0.8).

A

B

**S2 Figure. Manhattan, QQ and LD plots for IBD using the FarmCPU** **GWAS results.** On the Manhattan plot, the red dashed line is the Bonferroni-corrected significance threshold. Inflation factor (𝛌) is shown on the QQ plot.


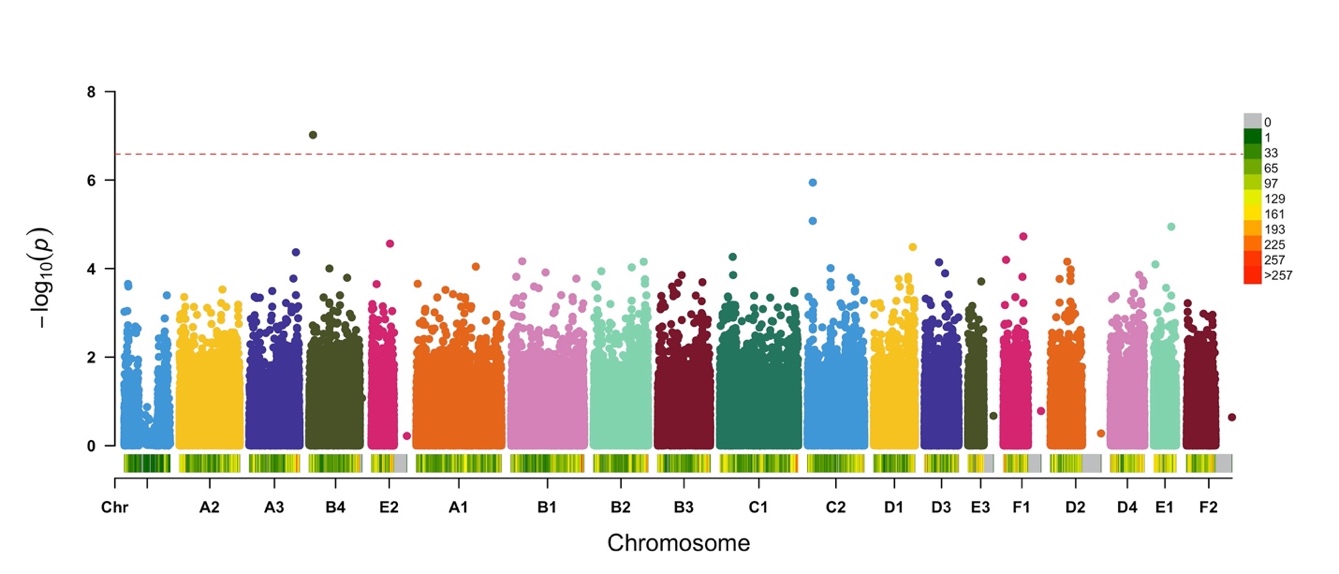


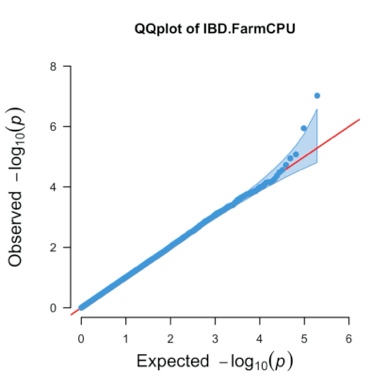


λ=1.03

**S3 Figure**. **Manhattan and QQ plots for diseases that did not reach genome-wide significance in the LMM GWAS.** On Manhattan plots, the red line is Bonferroni-corrected significance threshold, and the blue line is Bonferroni-corrected significance threshold calculated using unlinked SNPs. Inflation factors (𝛌) are shown on QQ plots.

(A) CKD


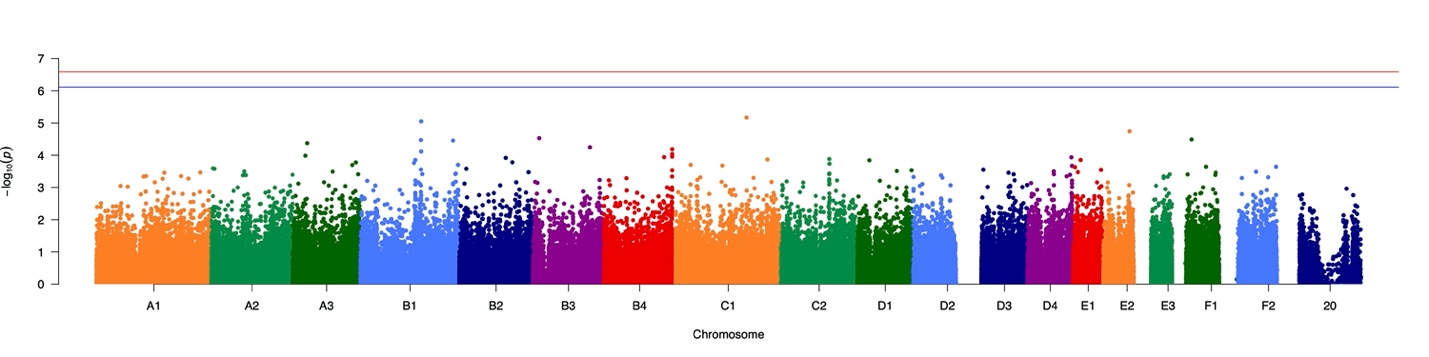


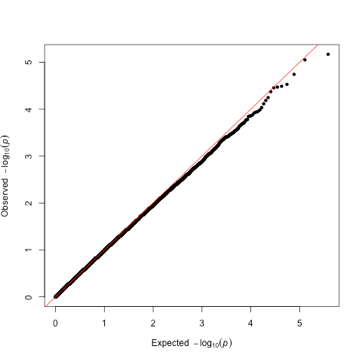


λ=1.00

(B) IBD


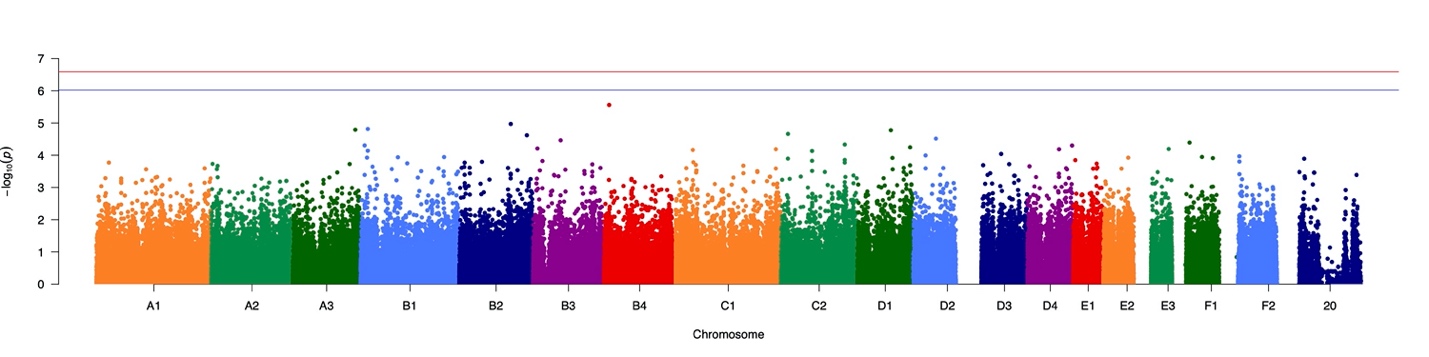


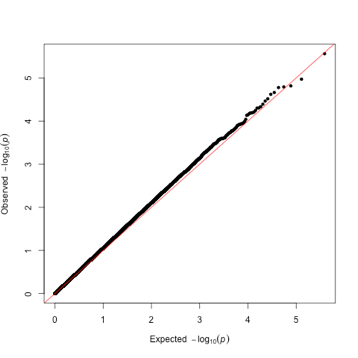


λ=1.04

(C) SCAL


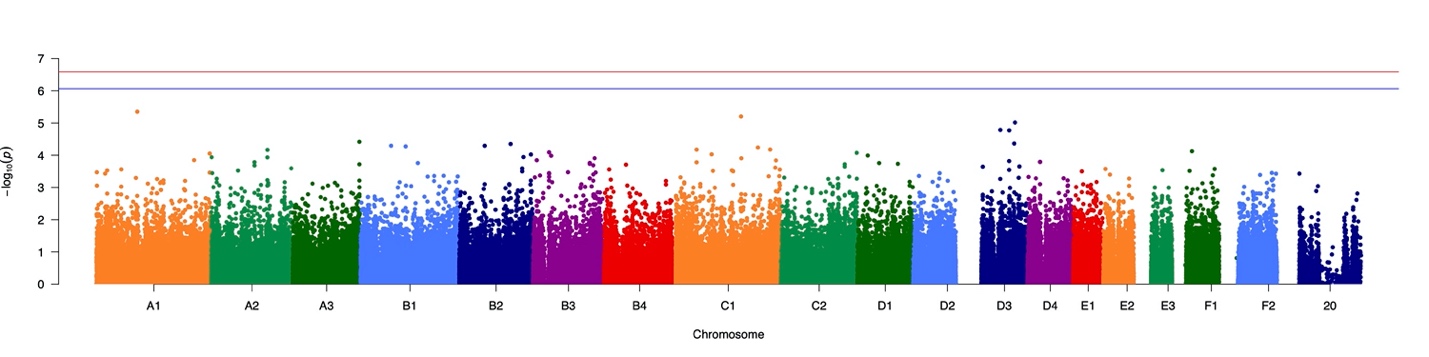


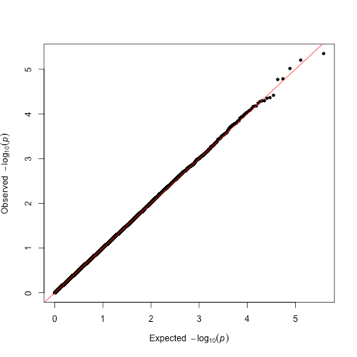


λ=1.00

(D) chronic enteropathy


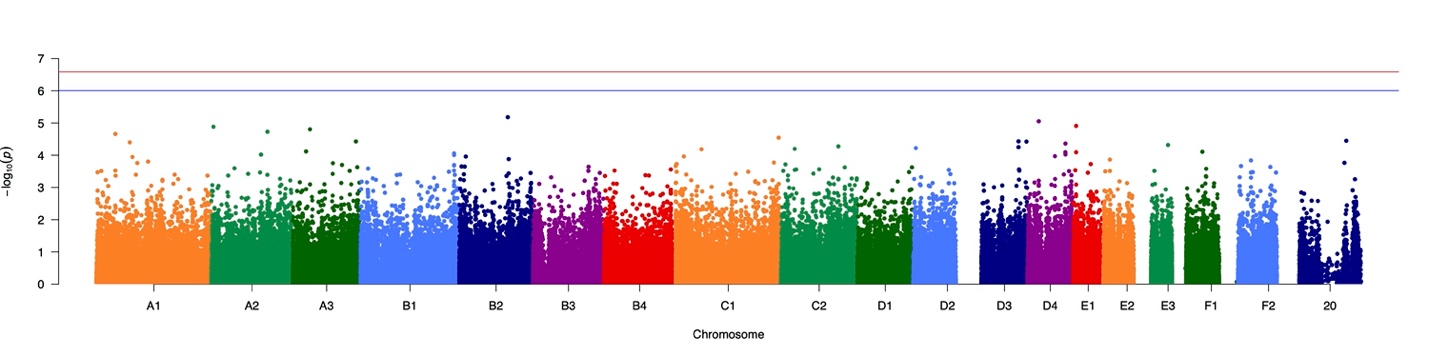


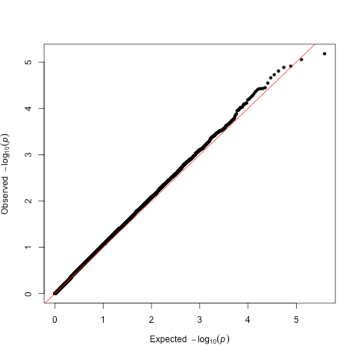


λ=1.05

(E) merged GI phenotypes


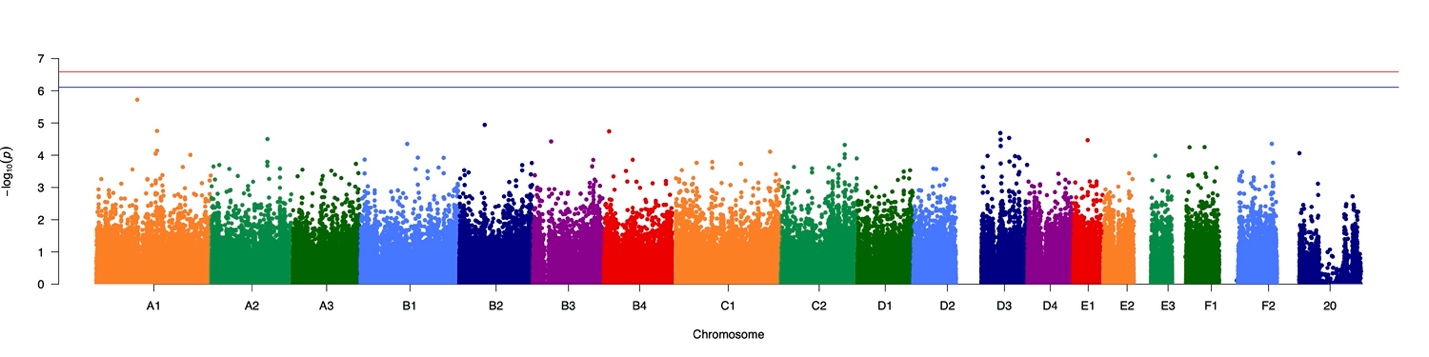

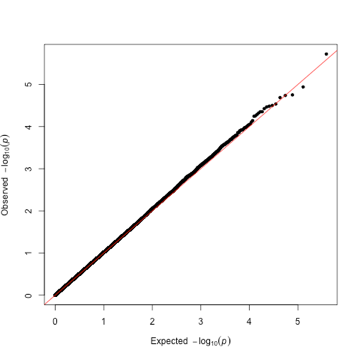


λ=1.02

**S4 Figure.** **Age distribution of cases and controls.** (A) HCM, (B) hyperthyroidism, (C) DM, (D) CKD, (E) chronic enteropathy, (F) IBD, (G) SCAL, (H) all GI, (I) FEK, (J) hypercalcemia. Age (in months) is shown on the X axis and number of cats is shown on the Y axis. Cases are the pink bars and controls are the blue bars.

(A) HCM. Note that there were 7 affected cats with an unknown date of birth that are not represented in the histogram.

(B) hyperthyroidism. Note that there were 2 affected cats with an unknown date of birth that are not represented in the histogram.

(C) DM. Note that there were 2 affected and 1 control cat with an unknown date of birth that are not represented in the histogram.


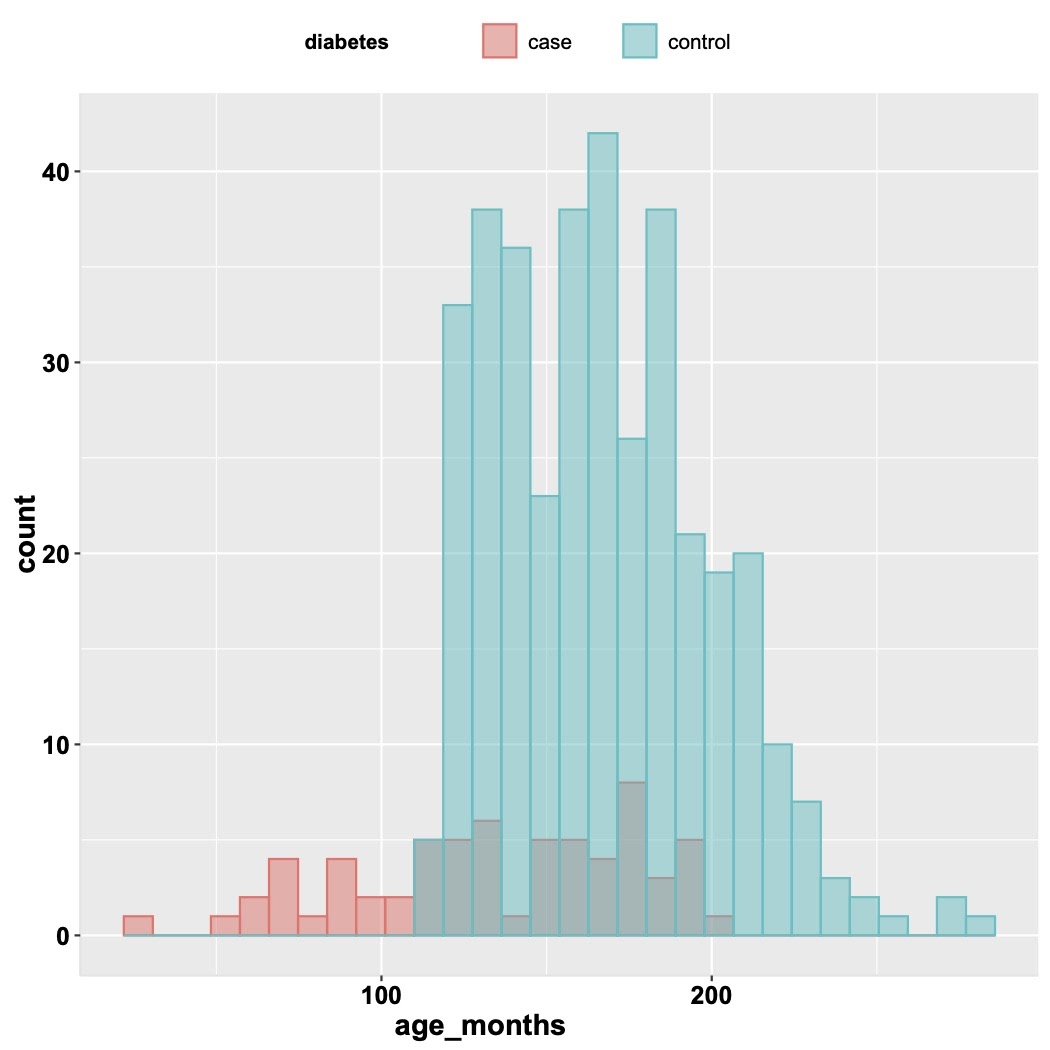


(D) CKD. Note that there were 2 affected cats with an unknown date of birth that are not represented in the histogram.

(E) chronic enteropathy

(F) IBD

(G) SCAL. Note that there was 1 affected cat with an unknown date of birth that is not represented in the histogram.

(H) all GI. Note that there was 1 affected cat with an unknown date of birth that is not represented in the histogram.

(I) FEK

(J) hypercalcemia. Note that there was 1 control cat with an unknown date of birth that is not represented in the histogram.


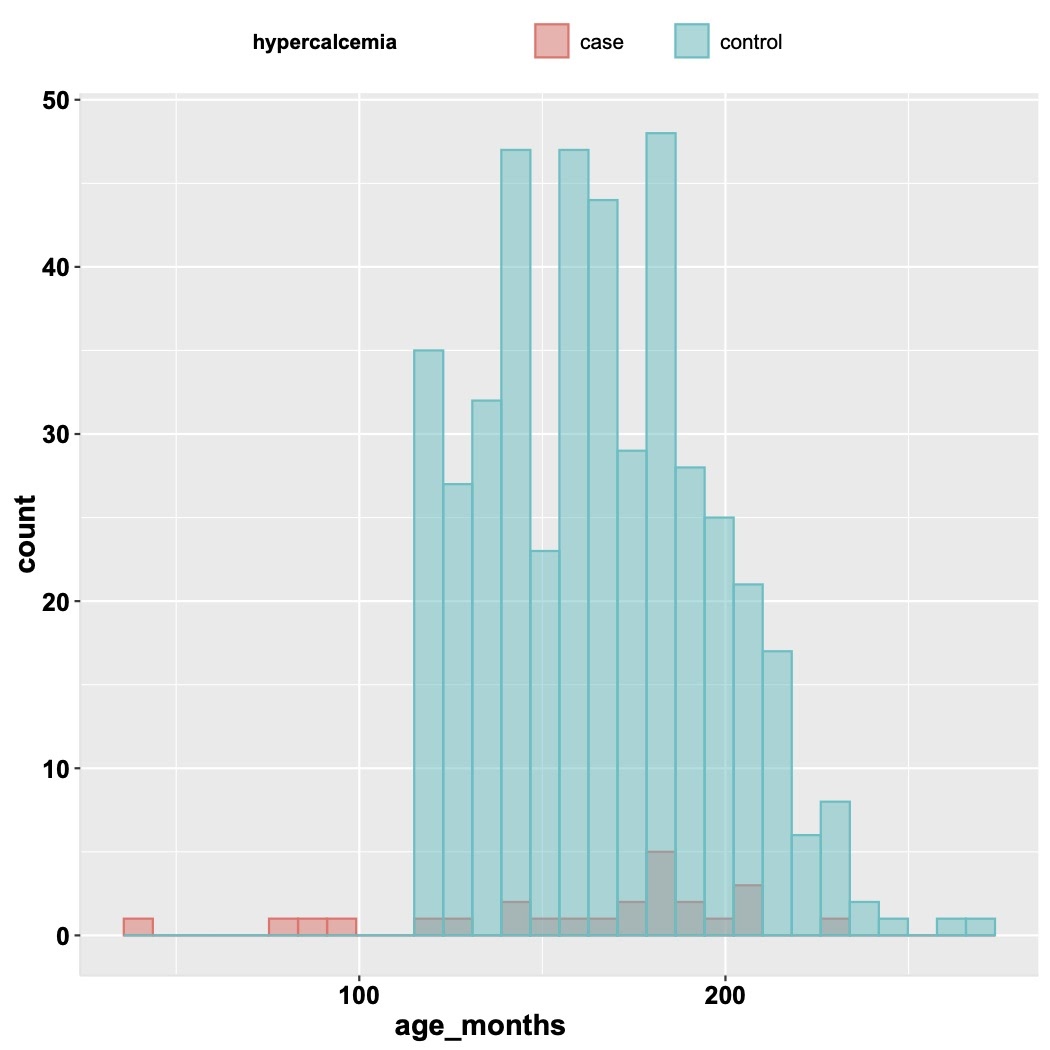


**S5 Figure.** **Weight distribution of cases and controls for DM.** Weight (in kg) is shown on the X axis and number of cats is shown on the Y axis. Cases are the pink bars and controls are the blue bars. Note that weight data was only available for 53 of the 67 cases, and 339 of the 366 controls used in the GWAS.

**
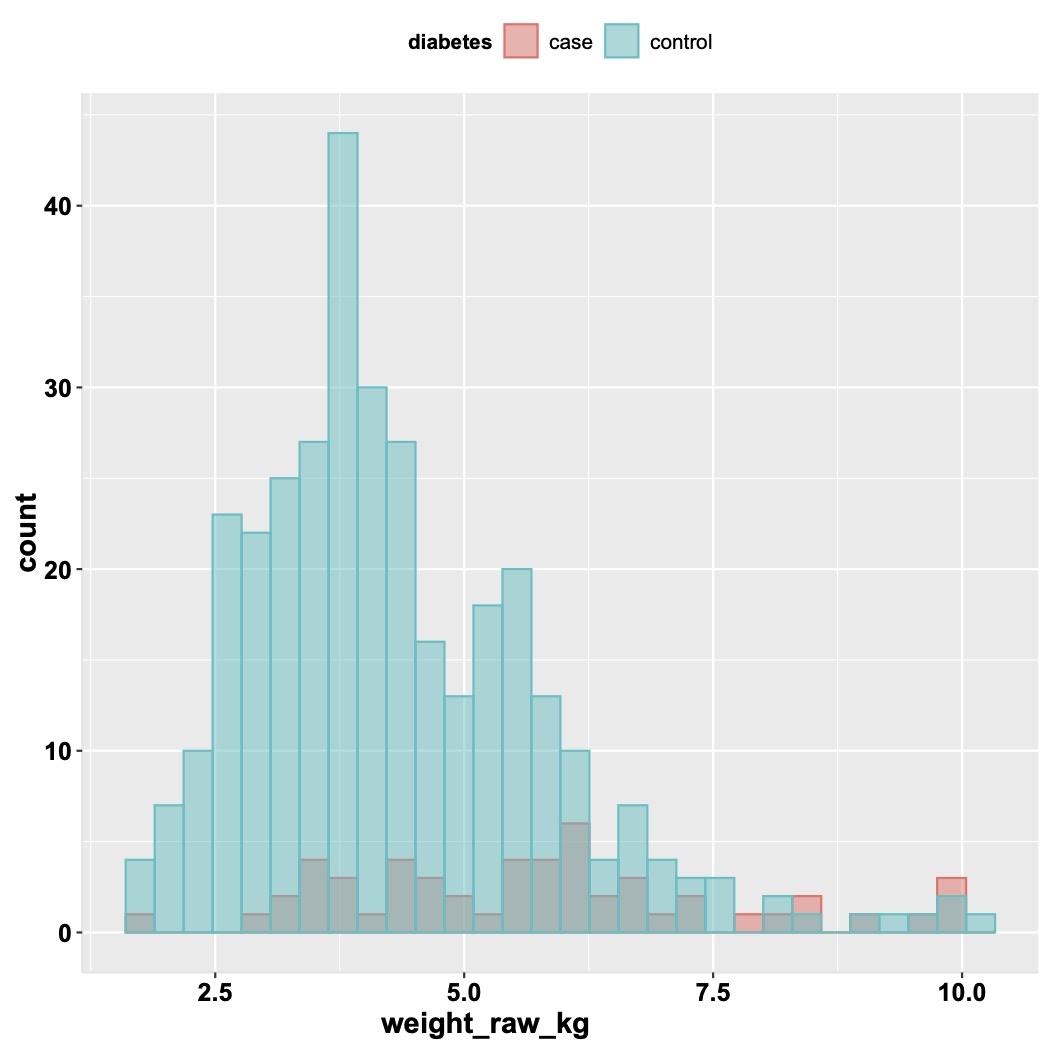
**
